# Sequential organ failure assessment score as a predictor of the outcomes of patients hospitalized for classical or exertional heatstroke

**DOI:** 10.1038/s41598-022-20878-1

**Published:** 2022-09-30

**Authors:** Kazuto Yokoyama, Tadashi Kaneko, Asami Ito, Yohei Ieki, Eiji Kawamoto, Kei Suzuki, Ken Ishikura, Hiroshi Imai, Jun Kanda, Shoji Yokobori

**Affiliations:** 1 Emergency and Critical Care Center, Mie University Hospital, 2-174 Edobashi, Tsu, 514-8507 Japan; 2 Department of Emergency Medicine, Teikyo University School of Medicine, Tokyo, Japan; 3 Department of Emergency and Critical Care Medicine, Nippon Medical School, Tokyo, Japan; 4 Japanese Association for Acute Medicine Heatstroke and, Hypothermia Surveillance Committee, Tokyo, Japan

**Keywords:** Medical research, Signs and symptoms

## Abstract

Heatstroke is a life-threatening event that affects people worldwide. Currently, there are no established tools to predict the outcomes of heatstroke. Although the Sequential Organ Failure Assessment (SOFA) score is a promising tool for judging the severity of critically ill patients. Therefore, in this study, we investigated whether the SOFA score could predict the outcome of patients hospitalized with severe heatstroke, including the classical and exertional types, by using data from a Japanese nationwide multicenter observational registry. We performed retrospective subanalyses of the Japanese Association for Acute Medicine heatstroke registry, 2019. Adults with a SOFA score ≥ 1 hospitalized for heatstroke were analyzed. We analyzed data for 225 patients. Univariate and multivariable analyses showed a significant difference in the SOFA score between non-survivors and survivors in classical and exertional heatstroke cases. The area under the receiver operating characteristic curve were 0.863 (classical) and 0.979 (exertional). The sensitivity and specificity of SOFA scores were 50.0% and 97.5% (classical), 66.7% and 97.5% (exertional), respectively, at a cutoff of 12.5, and 35.0% and 98.8% (classical), 33.3% and 100.0% (exertional), respectively, at a cutoff of 13.5. This study revealed that the SOFA score may predict mortality in patients with heatstroke and might be useful for assessing prognosis.

## Introduction

Heatstroke is a pathologic event that is internationally defined by the triad of hyperthermia, neurologic abnormalities, and exposure to hot weather or physical exertion. It affects people worldwide and is potentially hazardous due to increased risk of mortality. The causes of heatstroke are generally divided into classical causes, such as passive heatstroke in a hot environment, and exertional causes, which are associated with physical exercise. Regardless of cause, heatstroke is potentially life-threatening due to the risk of central nervous system dysfunction and multiple organ failure^1^.

Although some efforts have been made to recognize and define the severity of heatstroke, and hence predict the mortality and neurological status at discharge, there are still no internationally agreed definitions of the severity of heatstroke. Some reports have suggested that advanced age, disturbed consciousness, elevated serum creatinine (Cre) and total bilirubin (T-bil) levels, and coagulation disorder are risk factors for death^2,3^. In reports that focused on exertional heatstroke, acute kidney injury^4^ and myocardial injury^5^ were identified as risk factors. Other groups have also developed a scoring system (exertional heat stroke score [EHSS]) for classifying the severity of exertional heatstroke^6,7^.

Despite these efforts, a definitive tool for predicting the outcome of heatstroke that may be useful for assessing the prognosis has not yet been developed. Therefore, in this study, we evaluated a tool that may predict the outcome of heatstroke. In selecting the tool, we felt that the following factors were important when predicting mortality: (1) the tool should be available for classical and/or exertional heatstroke; (2) the tool could be used to assess heatstroke with multiple organ disfunction (we felt it was unnecessary to judge non-life-threatening cases); (3) the tool could use information recorded early, at the time of admission or hospitalization; and (4) the tool should be commonly available.

Internationally, the Sequential Organ Failure Assessment (SOFA) score is widely used to assess multiple organ dysfunction or failure as the main causes of death in critically ill patients^8^. Prior studies have suggested that the SOFA score is also useful for predicting mortality in patients with heatstroke^3–7,9,10^. However, those studies exhibited some weaknesses, including the use of populations comprising only exertional heatstroke, and single-center designs.

We therefore considered that the SOFA score could be a useful tool for assessing the risk of mortality in patients with severe classical or exertional heatstroke. Accordingly, we investigated the prognostic potential of the SOFA score using data from a Japanese nationwide multicenter observational registry collated annually by the Japanese Association of Acute Medicine (JAAM) (JAAM heatstroke registry).

Our hypothesis was that the early SOFA score predicts the mortality risk in both types of heatstroke, even in severe cases, and we tested it using the JAAM heatstroke registry.

## Methods

### Study design

In the present study, we performed retrospective analyses of data from the JAAM heatstroke registry, a nationwide multicenter observational registry collated annually since 2014 by the JAAM^11^. In 2019 (1 July–30 September), 148 hospitals participated in data collection (see acknowledgements). Patients were eligible for the registry if they were judged as having heat-related illness by the hospitals’ physicians and hospitalized. Anonymized medical data were entered into the registry using the JAAM online system. This registry database was mainly managed by Teikyo University Hospital. The registry protocol was approved by the ethics committee at Teikyo University Hospital. The participating hospitals also obtained approval from their ethics committees as necessary. And, this retrospective observational study was approved by ethics committees at Mie University Hospital, by using Ethical Guidelines for Medical and Biological Research Involving Human Subjects from Japanese government. Informed consent was obtained from all the study participants.

### Patients

The JAAM heatstroke registry is collated annually, and we used data recorded in 2019. Between 1 July and 30 September in 2019, a total of 734 patients were hospitalized for heatstroke and registered. For the present analyses, we retrieved data for patients with a SOFA score ≥ 1, excluding patients without multiple organ dysfunction in whom the SOFA score was not calculated. All of the patients were over 15 years old without intentional entry protocol.

### Study outcomes and statistical analysis

The eligible cases were divided into two groups of classical and exertional heatstroke, and statistical analysis were performed as follows. The following variables were retrieved retrospectively from the database: age, sex, outside onset, vital signs on admission (respiratory rate [RR], heart rate [HR], systolic and diastolic blood pressure [sBP and dBP], Glasgow Coma Score [GCS], and surface and core (rectum, bladder, esophagus, etc.) body temperature [sBT and cBT]), laboratory data on admission (pH, lactate, platelet count, prothrombin time [PT], T-bil, and Cre), Acute Physiology and Chronic Health Evaluation (APACHE) II score, SOFA score, and 28-day mortality. For this study, we used 28-day mortality as the outcome variable.

The patients were divided into two groups (non-survivor and survivors) based on their survival at 28 days. We compared the clinical data between these two groups using univariate and multivariable analyses. Univariate analyses were performed using the Mann–Whitney *U* test or Fisher’s exact test, as appropriate. Multivariable analyses were performed using logistic regression analysis, in which the dependent variable was 28-day mortality and the explanatory variables were age, sex (male), outside onset, RR, HR, sBP, dBP, cBT, pH, lactate, SOFA score, and APACHE II score. sBT was excluded from the analysis because of its correlation with cBT. GCS, platelet count, PT, T-bil, and Cre were also excluded because these are used to calculate the SOFA score. Receiver operating characteristic (ROC) curve analysis was performed to plot the SOFA score against 28-day mortality, and the area under the curve (AUC) was calculated. Sensitivity and specificity were calculated using the curve for individual cutoff values of the SOFA score.

In all analyses, a *P*-value of < 0.05 was considered statistically significant. All statistical analyses were performed with SPSS version 25.0 (IBM, Armonk, NY, USA).

### Ethical approval and consent to participate

The registry was approved by the ethics committees at Teikyo University and at the participating institutions and hospitals, as necessary. This retrospective observational study was approved by ethics committees at Mie University Hospital, by using Ethical Guidelines for Medical and Biological Research Involving Human Subjects from Japanese government. Informed consent was obtained from all the study participants.

## Results

**Figure 1:**
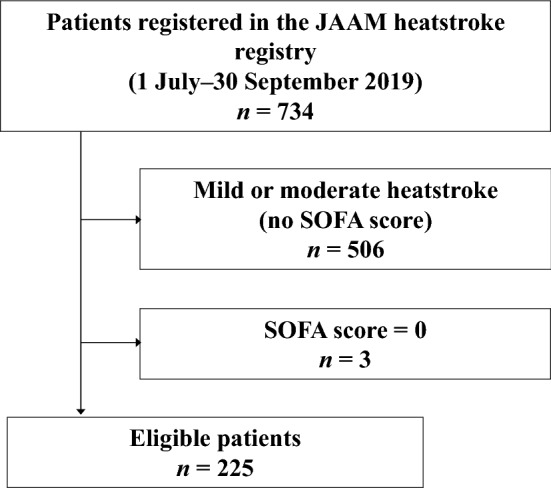
Patient disposition a total of 225 patients were considered eligible for this study after excluding patients with no SOFA score or a SOFA score of 0. JAAM, Japanese Association of Acute Medicine; SOFA, Sequential Organ Failure Assessment.

The registry comprised 734 patients, of which 225 met the inclusion criteria (i.e., SOFA score ≥ 1; Fig. 1). Therefore, the inclusion rate was 31% (225/734).

Table 1 shows the characteristics of the patients included in this analysis (all, classical, and exertional cases). The median age was 74 years, 72% of patients were male, 51% suffered heatstroke outside, and the median SOFA and APACHE II scores were 6 and 23, respectively. The survival status at 28 days was recorded in 146 of 225 patients (65%) and the 28-day mortality rate among these patients was 16% (24/146). Between classical and exertional cases, age, sex, outside onset, blood pressure, sBT, platelet, PT, and T-bil showed significant difference, and exertional cases shoed about 20 years younger age. Core body temperature was measured by 67% of bladder, 27% of rectum, 2% of esophagus, and etc.

**Table 1:** Characteristics of patients with heatstroke on admission.

Variables	All cases	Classical cases	Exertional cases	Univariate
	(*n* = 225)	(*n* = 151)	(*n* = 67)	*P* value
Age (years)	74 (57–84)	78 (69–86)	57 (44–77)	** < 0.001**
Sex (male)	161 (72%)	94 (62%)	63 (91%)	** < 0.001**
Outside onset	113 (51%)	51 (34%)	59 (86%)	** < 0.001**
RR (bpm)	26 (20–32)	27 (20–32)	24 (19–32)	0.333
HR (bpm)	116 (95–135)	118 (101–133)	106 (87–140)	0.131
sBP (mmHg)	124 (100–147)	131 (101–151)	114 (98–132)	**0.020**
dBP (mmHg)	75 (60–88)	78 (65–89)	67 (56–83)	**0.030**
GCS	11 (4–14)	11 (5–14)	11 (4–15)	0.177
sBT (°C)	38.9 (37.6–40.1)	39.1 (38.3–40.0)	38.0 (36.4–40.5)	**0.024**
cBT (°C)	39.8 (38.2–41.0)	39.9 (38.3–41.1)	39.8 (38.0–41.0)	0.535
pH	7.45 (7.40–7.49)	7.45 (7.40–7.50)	7.43 (7.39–7.49)	0.493
Lactate (mmoL/L)	2.9 (1.9–4.9)	2.8 (1.9–4.8)	3.2 (2.1–4.9)	0.439
Platelet count (10^4^/μL)	19.8 (14.3–24.1)	17.7 (13.4–23.0)	21.1 (18.0–27.7)	**0.001**
PT (%)	82 (64–96)	77 (62–93)	92 (75–99)	** < 0.001**
T-bil (mg/dL)	0.9 (0.7–1.4)	1.1 (0.7–1.6)	0.8 (0.6–1.3)	**0.006**
Cre (mg/dL)	1.4 (1.0–2.1)	1.3 (1.0–1.9)	1.5 (1.0–2.4)	0.054
SOFA score	6 (4–9)	6 (4–9)	6 (3–9)	0.174
APACHE II score	23 (15–29)	22 (16–29)	23 (12–29)	0.354
28-day mortality rate	24/146 (16%)	20/100 (20%)	3/43 (7%)	0.080

Values are median (interquartile range) or *n* (%) of patients.* P* value: classical cases v.s. exertional cases.

*RR* respiratory rate; *HR* heart rate; *sBP* systolic blood pressure; *dBP* diastolic blood pressure; *GCS* Glasgow Coma Scale; *sBT* surface body temperature; *cBT* core body temperature; *PT* prothrombin time; *T-bil* total bilirubin; *Cre* creatinine; *SOFA* Sequential Organ Failure Assessment; *APACHE* Acute Physiology and Chronic Health Evaluation.

Significant values are in bold.

Table 2 compares the clinical data between the non-survivors (*n* = 20) and survivors (*n* = 80) at 28 days in classical cases. The univariate analyses revealed significant differences between these two groups in terms of sBP, dBP, GCS, cBT, lactate, platelet, PT, Cre, SOFA score (*P* < 0.001), and APACHE II score. In the multivariable analysis, there were significant differences between the two groups in terms of sex and SOFA score (odds ratio [OR] 1.973 [95% confidence interval [CI] 1.050–3.706], *P* = 0.035).

**Table 2:** Univariate and multivariable comparisons between survivors and non-survivors at 28 days in classical cases.

Variables	Non-survivors (*n* = 20)	Survivors (*n* = 80)	Univariate *P* value	Multivariable *P* value	OR (95% CI)
Age (years)	63 (76–85)	79 (70–86)	0.330	0.502	0.967 (0.876–1.067)
Sex (male)	12 (60%)	53 (66%)	0.609	**0.039**	0.033 (0.001–0.843)
Outside onset	9 (45%)	22 (28%)	0.181	0.148	11.672 (0.420–324.5)
RR (bpm)	27 (22–35)	28 (21–32)	0.918	0.210	0.873 (0.706–1.080)
HR (bpm)	127 (100–135)	120 (102–137)	0.790	0.656	1.014 (0.955–1.077)
sBP (mmHg)	95 (82–118)	136 (107–154)	**0.001**	0.785	0.993 (0.943–1.045)
dBP (mmHg)	63 (49–81)	79 (66–90)	**0.010**	0.657	1.018 (0.941–1.101)
GCS	3 (3–8)	12 (8–14)	** < 0.001**	–	–
sBT (°C)	38.9 (38.4–40.4)	39.0 (38.3–40.0)	0.579	–	–
cBT (°C)	41.6 (39.8–42.0)	39.9 (38.4–40.9)	**0.004**	0.265	1.879 (0.619–5.704)
pH	7.43 (7.20–7.50)	7.46 (7.42–7.50)	0.184	0.401	0.03 (0.000–2595.8)
Lactate (mmoL/L)	5.0 (2.9–9.5)	2.6 (1.9–4.4)	** < 0.001**	0.855	1.054 (0.599–1.854)
Platelet (10^4^/μL)	12.0 (7.3–17.5)	18.0 (14.0–22.5)	**0.001**	–	–
PT (%)	62 (40–78)	81 (64–95)	**0.005**	–	–
T-bil (mg/dL)	1.0 (0.7–2.0)	1.2 (0.7–1.6)	0.786	–	–
Cre (mg/dL)	1.7 (1.5–2.1)	1.1 (0.9–1.7)	**0.001**	–	–
SOFA score	12 (8–15)	6 (4–8)	** < 0.001**	**0.035**	1.973 (1.050–3.706)
APACHE II score	33 (26–36)	20 (15–26)	** < 0.001**	0.568	0.922 (0.699–1.217)

Values are median (interquartile range) or *n* (%) of cases.

*OR* odds ratio; *CI* confidence interval; *RR* respiratory rate; *HR* heart rate; *sBP* systolic blood pressure; *dBP* diastolic blood pressure; *GCS* Glasgow Coma Scale; *sBT* surface body temperature; *cBT* core body temperature; *PT* prothrombin time; *T-bil* total bilirubin; *Cre* creatinine; *SOFA* Sequential Organ Failure Assessment; *APACHE* Acute Physiology and Chronic Health Evaluation.

Significant values are in bold.

Table 3 compares the clinical data between the non-survivors (*n* = 3) and survivors (*n* = 40) at 28 days in exertional cases. The univariate analyses revealed significant differences between these two groups in terms of GCS, PT, and SOFA score (*P* = 0.001). The multivariable analysis could not be calculated.

**Table 3:** Univariate and multivariable comparisons between survivors and non-survivors at 28 days in exertional cases.

Variables	Non-survivors (*n* = 3)	Survivors (*n* = 40)	Univariate *P* value	Multivariable *P* value	OR (95% CI)
Age (years)	49 (–)	57 (37–76)	0.603	–	–
Sex (male)	3 (100%)	36 (90%)	1.000	–	–
Outside onset	2 (67%)	35 (88%)	0.370	–	–
RR (bpm)	40 (–)	24 (20–30)	0.396	–	–
HR (bpm)	135 (–)	101 (87–139)	0.307	–	–
sBP (mmHg)	94 (–)	113 (100–138)	0.545	–	–
dBP (mmHg)	93 (–)	66 (56–80)	0.880	–	–
GCS	3 (–)	13 (6–15)	**0.024**	–	–
sBT (°C)	40.1 (–)	37.8 (36.4–39.7)	0.273	–	–
cBT (°C)	40.7 (–)	40.0 (38.1–41.1)	0.889	–	–
pH	7.46 (–)	7.42 (7.39–7.50)	0.982	–	–
Lactate (mmoL/L)	5.4 (–)	3.3 (2.1–4.9)	0.066	–	–
Platelet (10^4^/μL)	20.0 (–)	21.6 (16.3–28.8)	0.237	–	–
PT (%)	53 (–)	89 (72–99)	**0.048**	–	–
T-bil (mg/dL)	0.4 (–)	0.9 (0.6–1.3)	0.451	–	–
Cre (mg/dL)	2.7 (–)	1.5 (1.0–2.4)	0.218	–	–
SOFA score	12 (–)	6 (2–8)	**0.001**	–	–
APACHE II score	27.5 (–)	22 (12–28)	0.302	–	–

Values are median (interquartile range) or *n* (%) of cases.

*OR* odds ratio; *CI* confidence interval; *RR* respiratory rate; *HR* heart rate; *sBP* systolic blood pressure; *dBP* diastolic blood pressure; *GCS* Glasgow Coma Scale; *sBT* surface body temperature; *cBT* core body temperature; *PT* prothrombin time; *T-bil* total bilirubin; *Cre* creatinine; *SOFA* Sequential Organ Failure Assessment; *APACHE* Acute Physiology and Chronic Health Evaluation.

Significant values are in bold.

**Figure 2:**
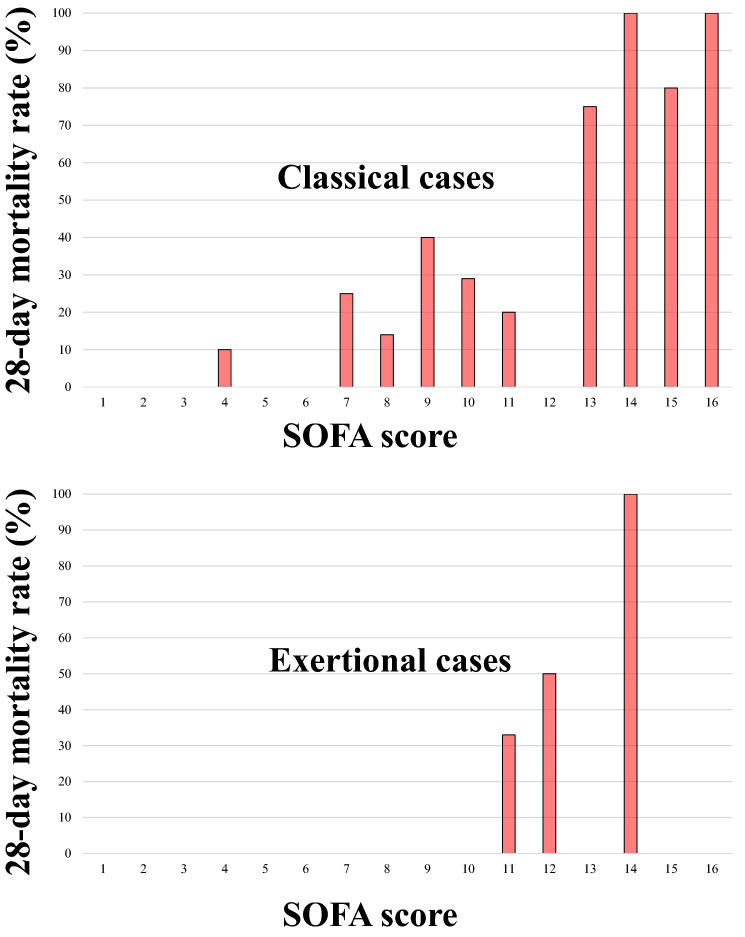
Bar graph of 28-day mortality rates according to SOFA scores in classical and exertional cases the mortality rate was correlated with the SOFA score, with a dramatic increase in mortality in patients with a SOFA score of ≥ 13 from both bar graphs. SOFA, Sequential Organ Failure Assessment.

Figure 2 shows a bar graph of the mortality rate according to the SOFA score in each classical and exertional cases. Refer to both bar graphs, these demonstrates a correlation between SOFA score and mortality rate, with a dramatic increase in the mortality rate in patients with a SOFA score ≥ 13.

**Figure 3:**
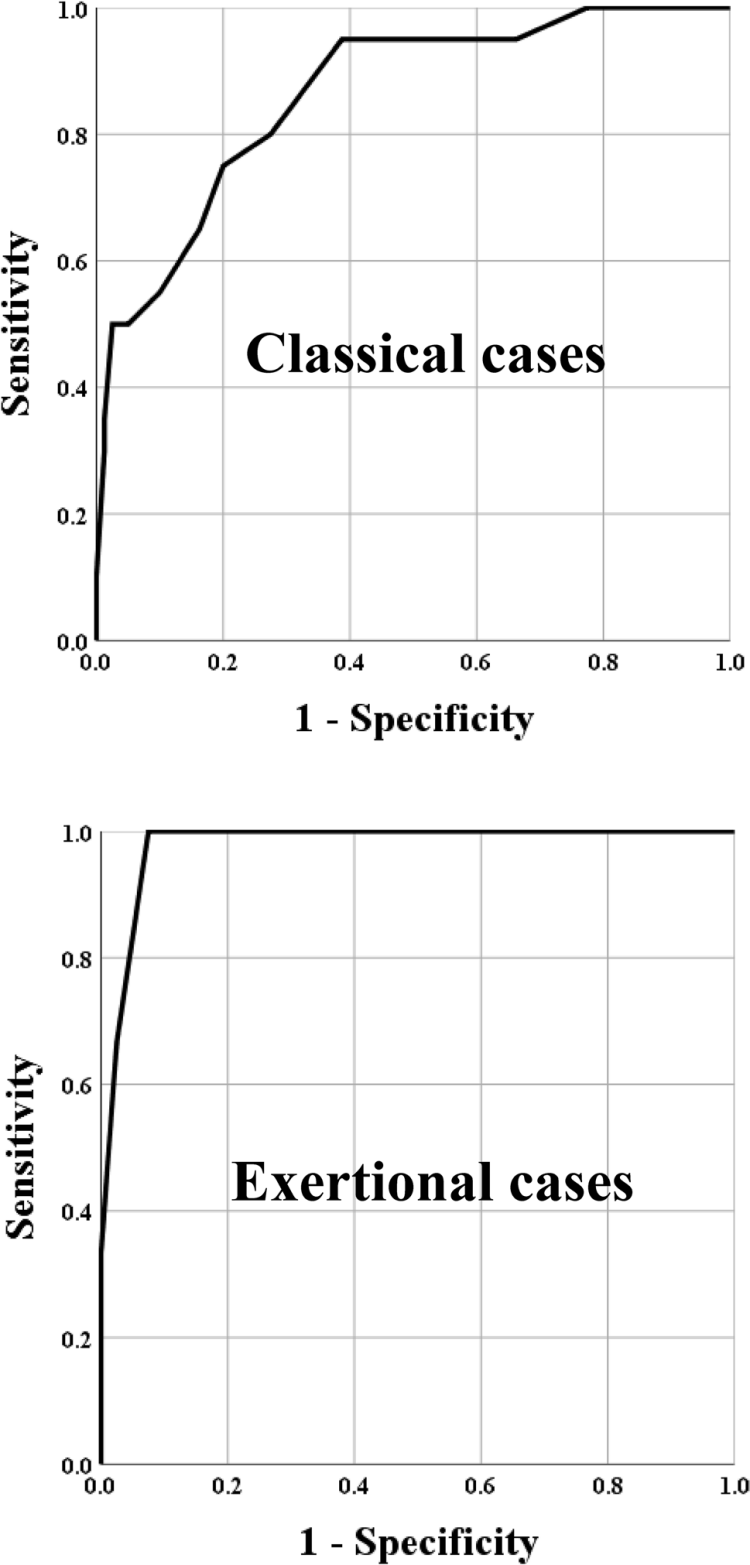
Receiver operating characteristic (ROC) curve analysis of SOFA score and mortality in classical and exertional cases The area under the curve ROC curve were 0.863 (*P* < 0.001) and 0.979 (*P* = 0.006), respectively. SOFA, Sequential Organ Failure Assessment.

Figure 3 and Table 4 show the results of the ROC analysis of SOFA score and 28-day mortality in classical and exertional cases. The AUC were 0.863 (*P* < 0.001) and 0.979 (*P* = 0.006), respectively. From both ROC analysis, at a SOFA score cutoff value of 12.5, both specificity was more than 95%, each the sensitivity and specificity were 50.0% and 97.5%, 66.7% and 97.5%, respectively, At the cutoff value of 13.5, each the sensitivity and specificity were 35.0% and 98.8%, 33.3% and 100.0%, respectively.

**Table 4:** Results of receiver operating characteristic analysis of SOFA scores for 28-day mortality in classical and exertional cases.

SOFA score cut-off value	AUC	Sensitivity	Specificity	*P* value
Classical (*n* = 100)	0.863 (0.775–0.951)			** < 0.001**
6.5		95.0%	46.2%	
8.5		75.0%	80.5%	
11.5		50.0%	95.0%	
12.5		50.0%	97.5%	
13.5		35.0%	98.8%	
Exertional (*n* = 43)	0.979 (0.936–1.000)			**0.006**
10.5		100.0%	92.5%	
11.5		66.7%	97.5%	
13.0		33.3%	100.0%	

*SOFA* sequential organ failure assessment; *AUC* area under the curve.

Significant values are in bold.

## Discussion

In the present study, univariate and multivariable analysis revealed a significant association between the SOFA score and 28-day mortality in each classical and exertional heatstroke patients hospitalized for multiple organ dysfunction. The ROC analysis of SOFA score and 28-day mortality was also significant with an AUC of 0.863 (*P* < 0.001) and 0.979 (*P* = 0.006), respectively.

Some reports have revealed the potential usefulness of the SOFA score^3–7,9,10^. Here, we have also shown an advantage of using SOFA score for predicting the outcome of patients included in the heatstroke database. The study population were hospitalized for classical or exertional heatstroke, and we eliminated patients without a SOFA score (in other words excluding mild and moderate cases). The results therefore suggest that the SOFA score may predict the outcome of heatstroke, particularly for severe cases.

Heatstroke is disease related to fever and, from a pathophysiological perspective, non-septic fever could show more rapid deterioration than sepsis-related fever. Nevertheless, the pathology of systemic damage and progression of multiorgan dysfunction are thought to overlap^12^. The SOFA score was previously shown to be useful for predicting in-hospital mortality in patients with sepsis^13^. Based on the present results, the SOFA score may be a reliable tool for assessing the prognosis of heatstroke, a non-septic fever, as demonstrated in patients with sepsis. Nevertheless, other factors may be needed in combination with the SOFA score to improve the accuracy of predicting the prognosis of heatstroke.

Other studies have sought to establish a new scoring scale for heatstroke. Yang et al. reported that the exertional heatstroke score (EHSS; based on body temperature, GCS, pH, lactate, PT, fibrinogen, troponin I, aspartate transaminase, T-bil, Cre, and acute gastrointestinal injury) showed potential for predicting the outcome of heatstroke and was evaluated in several studies^6,7^. Many of the parameters used in that EHSS score were also strong factors in our study. In fact, GCS, platelet, PT, and Cre differed significantly between non-survivors and survivors in univariate analyses. However, these variables are used to calculate the SOFA score and were therefore excluded from the multivariable analysis to avoid confounding. The ROC curve analyses in earlier studies showed superiority of the EHSS versus the SOFA score (EHSS score showed both sensitivity and specificity were more than 90%). However, if these tools are used for assessing prognosis, high sensitivity with 100% specificity (or at least 98%–99%) is needed. Therefore, the EHSS should be improved to increase sensitivity while retaining high specificity, which was < 50% in their study^6^. As tools for assessing prognosis, neither the EHSS nor the SOFA score displayed sufficient sensitivity.

To improve the predictive value of the SOFA score, lactate could be included as a candidate variable because it showed statistical significance in the univariate analysis. In fact, prior studies have already demonstrated the use of lactate for predicting mortality in critical care patients^14^, and a combination of lactate and SOFA score has been already applied in sepsis, with a pathology of shock and multiple organ dysfunction^15^. Accordingly, a combination of lactate and SOFA score is worth investigating in order to improve the predictive and prognostic value in heatstroke.

This study has several limitations. First, although we used data from a nationwide cohort, the study was performed retrospectively, which may introduce some bias. Second, the survival status at 28 days was only recorded for a proportion of patients (65%, 146/225), which may reduce data quality. Third, although we performed multivariable analysis, there might be residual confounding or other factors that may influence the results. Fourth, 28-day mortality was used as the outcome of interest in this study; the results may differ if we used 6-month or 1-year mortality. Fifth, our database did not have prehospital care information, there was a possibility that prehospital treatment might affect the outcome.

## Conclusions

These subanalyses of a Japanese nationwide multicenter observational heatstroke database using data from 2019 revealed that the SOFA score may be useful for predicting mortality and could be used to assess the prognosis of patients with severe heatstroke.

## Data Availability

The datasets used during the current study available from the corresponding author on reasonable request.

## Abbreviations

EHSS: Exertional heatstroke scale
SOFA: Sequential organ failure assessment
JAAM: Japanese association of acute medicine
HR: Heart rate
RR: Respiratory rate
sBP: Systolic blood pressure
dBP: Diastolic blood pressure
PT: Prothrombin time
T-bil: Total bilirubin
Cre: Creatinine
APACHE: Acute physiology and chronic health evaluation
ROC: Receiver operating characteristic
AUC: Area under the curve
OR: Odds ratio
CI: Confidence interval
